# Serum exosomal microRNA-34a as a potential biomarker in epithelial ovarian cancer

**DOI:** 10.1186/s13048-020-00648-1

**Published:** 2020-04-26

**Authors:** Kazuya Maeda, Hiroshi Sasaki, Shoko Ueda, Shunsuke Miyamoto, Shinichi Terada, Hiromi Konishi, Yuhei Kogata, Keisuke Ashihara, Satoe Fujiwara, Yoshimichi Tanaka, Tomohito Tanaka, Masami Hayashi, Yuko Ito, Yoichi Kondo, Takahiro Ochiya, Masahide Ohmichi

**Affiliations:** 1 Department of Obstetrics and Gynecology, Osaka Medical College, Osaka, Japan; 2 Department of Anatomy and Cell Biology, Osaka Medical College, Osaka, Japan; 3 Division of Molecular and Cellular Medicine, National Cancer Center Research Institute, Tokyo, Japan

**Keywords:** Serum exosome, miR-34a, Ovarian cancer, Biomarker

## Abstract

**Background:**

Ovarian cancer (OC) is a leading cause of cancer-related death in women, and thus an accurate diagnosis of the predisposition and its early detection is necessary. The aims of this study were to determine whether serum exosomal microRNA-34a (miR-34a) in ovarian cancer could be used as a potential biomarker.

**Methods:**

Exosomes from OC patients’ serum were collected, and exosomal miRNAs were extracted. The relative expression of miR-34a was calculated from 58 OC samples by quantitative real-time polymerase chain reaction.

**Results:**

Serum exosomal miR-34a levels were significantly increased in early-stage OC patients compared with advanced-stage patients. Its levels were significantly lower in patients with lymph node metastasis than in those with no lymph node metastasis. Furthermore, its levels in the recurrence group were significantly lower than those in the recurrence-free group.

**Conclusions:**

Serum exosomal miR-34a could be a potential biomarker for improving the diagnostic efficiency of OC.

## Background

Ovarian cancer (OC) is a leading cause of cancer-related death in women and the most lethal gynecologic malignancy. The keystone for improving health outcomes is the early detection and accurate diagnosis of the disease. Despite the emergence of novel therapies such as antiangiogenics in recent years, little improvement in survival has been achieved. At present, the standard therapy for ovarian cancer is cytoreductive surgery and postoperative adjuvant chemotherapy with platinum-based compounds. However, despite these treatments, the 5-year overall survival rates for OC are reported to be 46.0% for stage III and 25.1% for stage IV [1]. A poor prognosis can be expected due to the asymptomatic nature of this disease [2]. Such outcomes highlight the urgency to establish early diagnostic methods for OC with novel, more effective, and less invasive approaches. One feasible approach for diagnosing ovarian cancer at an early stage may involve the identification of useful and non-invasive potential biomarkers.

MicroRNAs (miRNAs) consist of 18–25 nucleotides and are highly conserved. It is well-known that mature miRNAs regulate gene expression by binding to complementary nucleotide sequences in 3′ untranslated regions (UTRs) of target messenger RNAs (mRNAs) [3]. Increasing numbers of advanced studies reveal important roles of miRNAs in various tumors, where they regulate several processes, such as cell differentiation, proliferation, and apoptosis [4, 5].

Several studies have shown that miR-34a is usually downregulated and suppresses tumor migration and invasion in various tumors, and inhibits c-Met expression in human hepatocellular carcinoma cells [6]. Decreased expression of miR-34a and increased expression of HDAC1 are closely related to OC cell development, thus miR-34a may function as a tumor repressor by directly targeting and modulating HDAC1 expression in OC cells.

To explain the stability of circulating RNAs, it was suggested that extracellular RNAs are included within lipoprotein vesicles. Indeed, exogenous RNAs added to plasma or blood are immediately degraded, whereas endogenous plasma RNAs are stable for hours under the same conditions [7]. Furthermore, treatment with some detergents results in the immediate degradation of plasma extracellular RNAs, apparently due to the disruption of the lipid vesicles. These findings clearly indicate that extracellular RNAs are packaged in secretory particles such as apoptotic bodies or exosomes, leaving them protected from ubiquitous ribonucleases.

Exosomes have been postulated to have an important role in cell–cell communication and appear to affect target cells either by stimulating them directly through ligands expressed on the cell or by transferring molecules between cells. Ratajczak et al. [8] demonstrated the presence of exosomal RNA and provided evidence for the horizontal transfer of genetic information between cells. Since there is a possibility that exosomes of detected in plasma contain tumor cell-specific miRNA, their use as potential biomarkers of disease progression might be a reasonable option. There have been some reports at the miRNA level of exosomes isolated from OC tissue or ascites [9–15]. However, to our knowledge, there are few reports at the miRNA level of exosomes isolated from the plasma of OC patients [16–19].

The present study investigated miRNA levels in exosomes isolated from the plasma of OC patients with different clinical presentations upon diagnosis, and retrospectively evaluated the relationship between these findings and disease outcome.

## Materials and methods

### Study populations

Blood samples were collected from 58 epithelial OC patients directly before surgery from 2013 to October 2017. The patients were treated according to the national guidelines at Osaka Medical College, Department of Gynecology, and histologically confirmed to be International Federation of Gynecology and Obstetrics (FIGO) stages I–IV.

Blood collection and experiments were performed in compliance with the Helsinki Declaration and were approved by the institution’s ethics committee. The experiments were undertaken with the understanding and written informed consent of each subject. Regarding blood processing, uniform management concerning the specific protocols was performed.

### Isolation of total exosomes from plasma

Blood (10 ml) from OC patients was collected in a serum separator tube and processed within an hour. Separation of the serum was accomplished by centrifugation at 3000×*g* for 30 min to remove cells and cell debris. The cell-free serum samples were stored in aliquots at − 80 °C. The samples were then ultracentrifuged at 100,000×*g* for 240 min at 4 °C using a Beckman™ L-90 K ultracentrifuge (Brea, CA, USA), and then the pellets were washed with phosphate-buffered saline (PBS). The exosome samples were stored at − 80 °C for later analysis.

### Electron microscopy

Exosome pellets were resuspended in PBS, and the solution was dropped onto a carbon-coated copper grid with a mesh diameter of 2 nm for 2 min. The excess liquid was removed, and filter paper was used to drain the grid; a drop was negatively stained with phosphotungstic acid and loaded onto the grid for 5 min. The grid was then dried at room temperature. Finally, the samples were observed by transmission electron microscopy as previously described [20].

### Western blotting analyses

The exosomal samples were plated onto six-well plates and lysed with radioimmunoprecipitation assay buffer (RIPA buffer; 25 mM Tris-HCl pH 7.6, 150 mM NaCl, 1% sodium deoxycholate, 1% NP-40, and 0.1% sodium dodecyl sulfate). Lysates were separated by 5–20% sodium dodecyl sulfate-polyacrylamide gel electrophoresis and transferred to polyvinylidene difluoride membranes, followed by incubation with primary antibodies (CD63) and then incubation with the corresponding secondary horseradish peroxidase-conjugated IgG. The proteins were visualized with an electrochemiluminescent system (PerkinElmer Life Science, Waltham, MA, USA).

### Extraction of exosomal miRNAs

Total miRNAs were extracted from exosomes resuspended using the miRVana™ miRNA Isolation Kit (#AM1560; Life Technologies, Carlsbad, CA, USA) according to the manufacturer’s recommendations.

### Quantitative reverse transcription polymerase chain reaction (qRT-PCR) of miR-34a from serum exosomal microRNA

MiRNA qRT-PCR was performed using the StepOnePlus Real-Time PCR System (Applied Biosystems, Foster City, CA, USA). Total RNA was transcribed into cDNA using the TaqMan MiRNA Reverse Transcription Kit (#4366596; Applied Biosystems). Mature miR-34a was assayed using the TaqMan assay. To normalize the miRNA expression, RNU48 was used as an endogenous control for cellular miRNA. Each qRT-PCR assay was performed in triplicate, and the relative expression of miR-34a was calculated using the 2^-ΔΔCt^ method.

### Quantitative reverse transcription polymerase chain reaction (qRT-PCR) of miR-34a from ovarian cancer tissue or cell lines

To clarify miR-34a derived from ovarian cancer itself, we performed qRT-PCR of *miR-34a* from stage I ovarian cancer tissue samples (serous, endometrioid, and clear cell carcinoma) and ovarian cancer cell lines (CAOV3, mucinous carcinoma; A2780, serous carcinoma; and RMG-1, clear cell carcinoma). Total miRNA was extracted from these tissue samples or cell lines following their resuspension using the miRVana™ miRNA Isolation Kit. Next, miRNA qRT-PCR was performed using the StepOnePlus Real-Time PCR System as above.

## Results

### Verification of exosomes

**Fig. 1:**
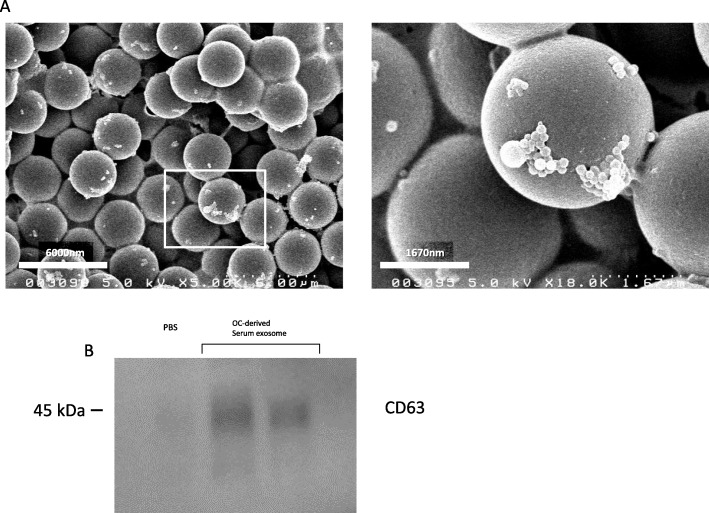
Verification of exosomes. **a** Transmission electron microscopy revealed that the clusters isolated from serum were round or oval membrane vesicles largely between 30 and 100 nm in size and were homogeneous in appearance. **b** Western blotting revealed that the specific exosomal protein marker CD63 was expressed in isolated serum exosomal pellets as specific bands

We first confirmed whether exosomes were present in the isolated serum pellets by ultracentrifugation. Transmission electron microscopy revealed that the clusters isolated from serum were round or oval membrane vesicles of predominantly 30 to 100 nm in size and were homogeneous in appearance (Fig. 1a), showing the characteristic appearance of exosomes. We next examined the expression of CD63, which is a specific exosomal protein marker [21]. The lysates of the isolated serum pellets were subjected to western blotting with anti-CD63 antibody. The compatible band for CD63 was detected as a specific band (Fig. 1b), suggesting the expression of CD63. These results suggest the successful extraction of serum exosomes.

### Elevated serum exosomal miR-34a in early-stage OC patients

**Fig. 2:**
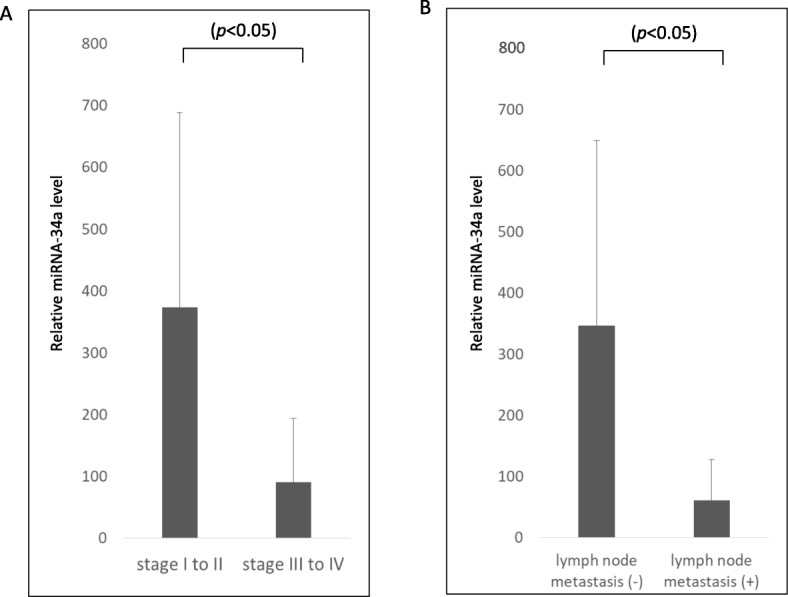
**a** The relative expression of miR-34a in early-stage OC patients (stage I or II) was significantly higher than that in advanced-stage patients (stage III or IV). **b** The serum exosomal miR-34a level was significantly lower in patients with lymph node metastasis than in those with no lymph node metastasis

The relative expression of miR-34a in serum exosomes was calculated among the OC patients. A total of 58 sera samples were collected. The median follow-up time was 52 months (range, 38–74 months). The mean age of the OC patients was 57.9 years (range, 34–76 years). The patients’ clinical characteristics and information are listed in Table 1. Real-time relative quantification was performed to investigate serum exosomal miRNA. We first examined the difference in the relative serum exosomal expression of miR-34a between early-stage and advanced-stage OC patients. The relative level of serum exosomal miR-34a in early-stage OC patients (stage I to II) (*n* = 25) was significantly higher than that in advanced-stage OC patients (stage III to IV) (*n* = 28) (*P* < 0.05) (Fig. 2a). Next, we examined the difference in the relative expression of serum exosomal miR-34a by lymph node metastasis. The relative level of serum exosomal miR-34a was significantly higher in patients with no lymph node metastasis (*n* = 37) compared with those with lymph node metastasis (*n* = 21) (*P* < 0.05) (Fig. 2b). These data suggest that the relative level of serum exosomal miR-34a in early-stage OC patients appears to be higher than that in advanced-stage OC patients.

**Table 1:** Patients’ characteristics

	Epithelial ovarian cancer (*n* = 58)	
Age, years: median (range)	57.9 (34–76)	
Histologic type	Serous	27 (47%)
	Clear	15 (26%)
	Endometrioid	6 (10%)
	Mucinous	6 (10%)
	Others	4 (7%)
FIGO Stage	I	21 (36%)
	II	5 (9%)
	III	20 (34%)
	IV	12 (21%)

### Association of serum exosomal miR-34a level and cancer recurrence

**Fig. 3:**
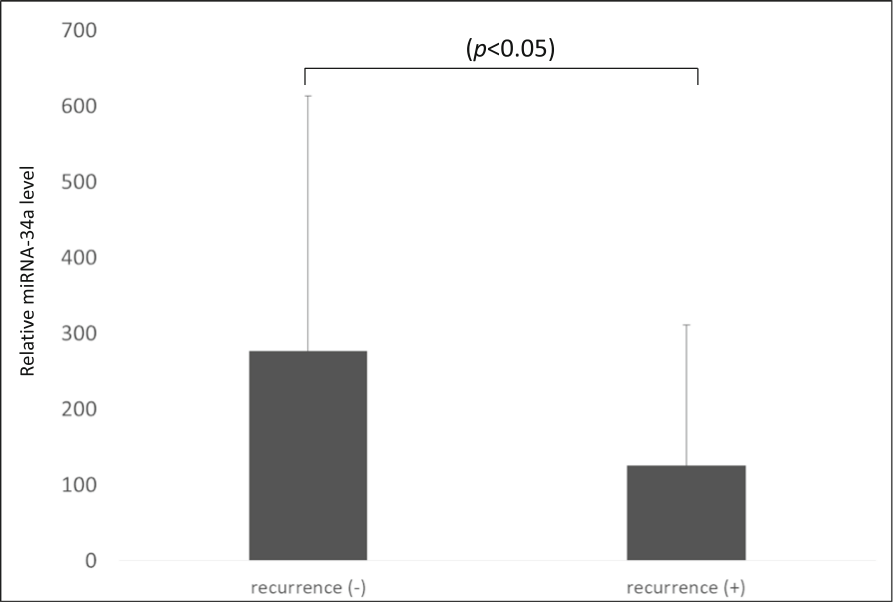
The relative level of miR-34a in the recurrence-free group was significantly higher than that in the recurrence group

We also investigated whether circulating levels of exosomal miR-34a were related to recurrence in OC patients. The relative level of serum exosomal miR-34a was compared between the group with recurrence within 3 years of primary surgery (*n* = 21) and the group that remained recurrence-free for 3 years (*n* = 37). The relative level of serum exosomal miR-34a in the recurrence-free group was significantly higher than that in the recurrence group (*P* < 0.05) (Fig. 3).

### Differences in serum exosomal miR-34a profile by stage and histological type

**Fig. 4:**
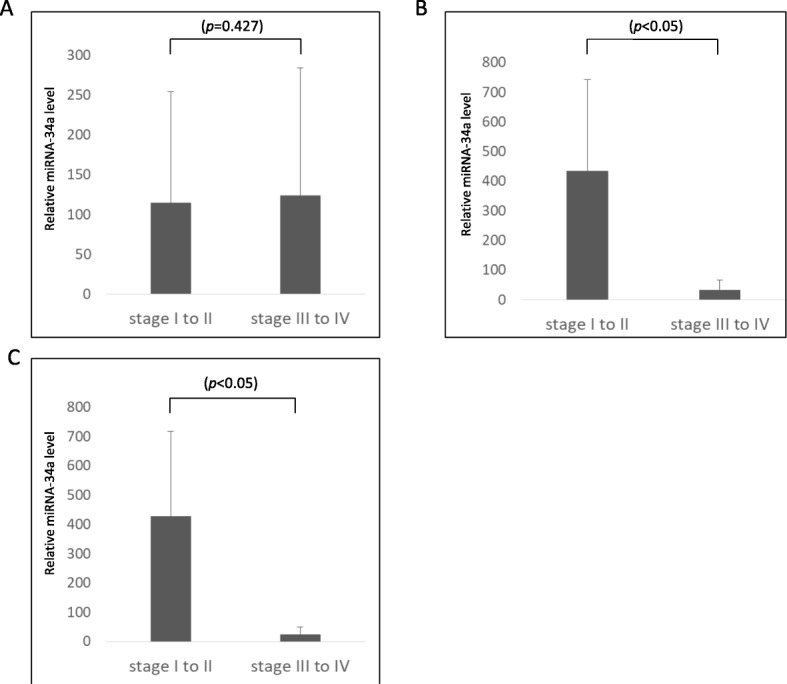
**a** In serous carcinoma, the relative level of miR-34a showed no significant difference between the early-stage OC group and the advanced-stage OC group. **b** In non-serous carcinoma, the relative level of miR-34a in the early-stage OC group was significantly higher than that in the advanced-stage OC group. **c** In clear cell carcinoma, the relative level of *miR-34a* in the early-stage OC group was significantly higher than that in the advanced-stage OC group

We next examined whether the profile of serum exosomal miR-34a differed among histological types (serous, clear-cell, endometrioid, and mucinous carcinoma). In serous carcinoma (*n* = 22), the relative level of serum exosomal miR-34a was not significantly different between the early-stage OC group (*n* = 5) and advanced-stage OC group (*n* = 17) (*P* = 0.427) (Fig. 4a). In contrast, in non-serous carcinoma (*n* = 30), the relative level of serum exosomal miR-34a in the early-stage group (*n* = 21) was significantly higher than that in the advanced-stage group (*n* = 9) (*P* < 0.05) (Fig. 4b). Furthermore, in the cases of clear cell carcinoma (*n* = 15), the relative level of serum exosomal miR-34a in the early-stage group (*n* = 10) was significantly higher than that in the advanced-stage group (*n* = 5) (*P* < 0.05) (Fig. 4c).

### Correlation of serum exosomal miR-34a levels with serum CA125 levels

**Fig. 5:**
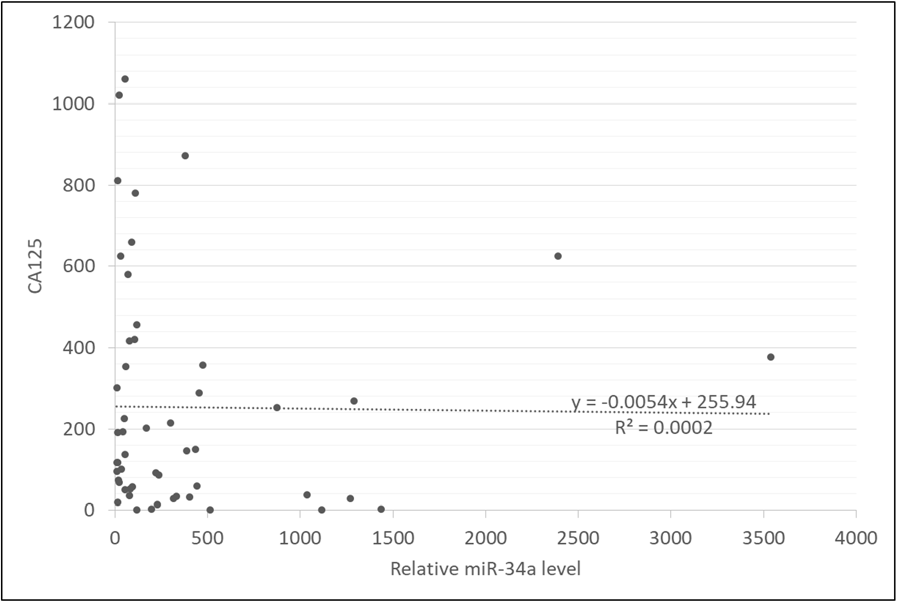
There was no correlation between the relative serum exosomal miR-34a level and the serum CA125 level

Serum CA125 is best known as a biomarker to monitor epithelial ovarian cancer and for the differential diagnosis of pelvic masses according to previous reports [9, 22, 23]. Serum levels of CA125 are routinely monitored in patients with OC, and an increase from an individualized nadir concentration is a prognostic indicator of cancer recurrence [24]. Therefore, we examined whether the level of serum exosomal miR-34a was correlated with that of serum CA125. No significant correlation was detected (*n* = 52) (R = 0.0002) (Fig. 5).

### Differences in miR-34a profiles by histological type in ovarian cancer tissue and cell lines

**Fig. 6:**
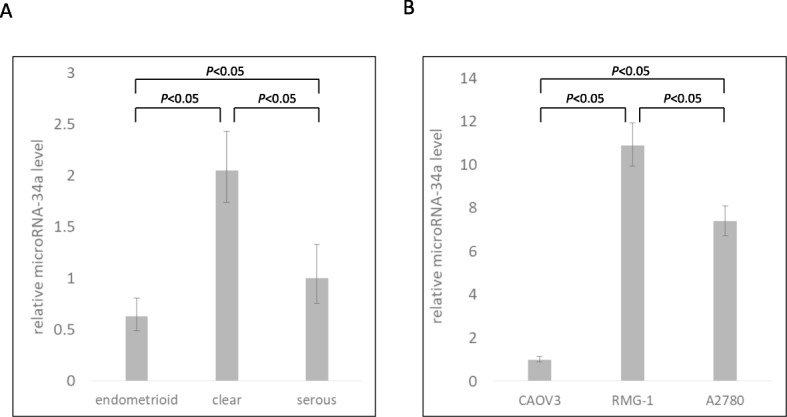
**a** The relative level of tissue miR-34a in clear cell carcinoma was significantly higher than that in serous carcinoma or mucinous carcinoma. **b** The relative miR-34*a* level in clear cell carcinoma RMG-1 cells was significantly higher than that in CAOV3 (mucinous carcinoma) or A2780 (serous carcinoma) cells

We examined whether the profile of miR-34a differed among histological types. The relative level of miR-34a in clear cell carcinoma tissue was significantly higher than that in serous or endometrioid carcinoma tissue (Fig. 6a). Furthermore, we examined the relative level of miR-34a in different types of human ovarian cancer cell lines. The relative miR-34a level in RMG-1 (clear cell carcinoma) cells was significantly higher than that in CAOV3 (mucinous carcinoma) or A2780 (serous carcinoma) cells (Fig. 6b).

## Discussion

In women, OC has the fifth highest mortality rate among malignant tumors. To diagnose and treat OC at an early stage and thus obtain a good prognosis, the identification of useful and non-invasive screening methods is important.

In recent years, numerous studies have confirmed the potential utility of miRNA in tumor diagnosis and prognosis assessment [10]. MiR-34a detected in plasma has been proposed as a biomarker in breast cancer [25] and lung cancer [26]. It was reported that a lot of miRNA are deferentially expressed in OC. Deb et al. summarized the current knowledge regarding the role of miRNA expression in OC [27]. In addition, several previous studies have shown that stable circulating miRNAs play an increasingly important role in the diagnosis of OC [9–15]. However, to our knowledge, few reports have described miRNA levels in exosomes isolated from the plasma of OC patients [16–19]. Tissue miRNAs present poor stability, but exosomal miRNAs in serum are thought to be more stable [28]. Therefore, we examined whether serum exosomal miR-34a serve as a potential biomarker of OC.

We focused on miRNA in serum exosomes, as exogenous miRNA added to plasma or blood is immediately degraded. Exosome are highly enriched in tetraspanins, a protein superfamily that organizes membrane microdomains [29–31]. We detected CD63 from isolated exosomes. CD63 encodes a protein that is a member of the tetraspanin family. Therefore, the expression of CD63 indicates the existence of exosomes. As we demonstrated, transmission electron microscopy revealed that the clusters isolated from serum were round or oval membrane vesicles largely between 30 and 100 nm in size and were homogeneous in appearance, suggesting that these vesicles were exosomes. Previous studies have also shown the significance of detecting exosomes using immunoblotting against CD63 and electron microscopy [16, 21, 32].

Exosomes derived from cancer contain tumor cell-specific miRNA, and hence they may be useful as potential biomarkers of disease progression. However, few reports have focused on serum exosomal miRNA [16–19], and thus this study may include some unique findings or interpretations. Interestingly, the analysis of the relative expression of serum exosomal miRNA in our study revealed that early-stage OC patients had significantly higher levels of exosomal miR-34a than advanced-stage patients. MiR-34a is usually downregulated and suppresses tumor migration and invasion by inhibiting c-Met expression [6]. A previous study showed that high c-Met expression is associated with lymph node metastasis and poor prognosis [33]. The decreased expression of miR-34a and the increased expression of HDAC1 are closely related to OC cell development, and thus miR-34a may function as a tumor repressor by directly targeting and modulating HDAC1 expression in OC cells. Therefore, miR-34a causes suppression of lymph node metastasis or tumor recurrence.

Our study shows the relative level of serum exosomal miR-34a was not significantly different between the early and advanced stages of serous carcinoma, while it was significantly different in non-serous carcinoma, especially clear cell carcinoma. There is evidence that p53, which acts as a tumor suppressor, transactivates the miR-34 family [34, 35]. These miRNAs interfere with the mRNA of crucial cellular proliferative and anti-apoptotic regulators and negatively control their expression, thus supporting cell cycle arrest, senescence, and apoptosis. The tumor-suppressing properties of this p53–miR34 interplay are of special importance during p53-detected DNA damage. Consecutive mutation of TP53 may favor carcinogenesis and tumor proliferation by reducing the levels of intracellular miR-34 family members. TP53 mutation represents the driver mutation in more than 95% of high-grade serous cancer cases, but is very uncommon in non-serous carcinoma, such as clear cell carcinoma [36, 37]. We postulate that this difference in TP53 mutation may correlate with the expression of miR-34a.

Tumor biomarkers are a focus of tumor research, and recent studies have identified the potential of various tumor biomarkers in gynecological oncology in addition to their diagnostic application. For example, the expression of p16 INK4a protein has been reported as a progression/regression tumor marker in LSIL cervix lesions [38, 39]. Other studies reported that combinations including a five-DNA methylation panel [40], a circulating miRNA panel [12], a serum miRNA and CA125 panel [41], and CPG island methylation [38] are of great significance in tumor diagnostic efficacy, prognosis, surgical guidance, and epigenetic therapy. In our study, Fig. 5 shows that there was no significant correlation between CA125 and miR-34a levels, indicating that the measurement of miR-34a may function as an independent biomarker of OC from CA125. It is well-known that CA125 is most commonly used as a conventional tumor marker of OC, but it is elevated not only in ovarian cancer but also in benign ovarian cysts, including ovarian endometriotic cysts and luteal cysts, and endocrine, digestive, and nutritional metabolic diseases [16]. CA125 is also known to be elevated during menstruation [42]. Thus, CA125 is non-specific biomarker for OC. Therefore, our results demonstrating that CA125 is not correlated with miR-34a suggest that serum exosomal miR-34a could serve as a potential biomarker of OC in the clinic. Su et al. reported that serum exosomal miR-375 and miR-1307 enhance the diagnostic power of CA125 [16]. CA125 and miR-34a may function as complimentary biomarkers of OC.

Serum exosomal miR-34a has great potential utility as a prognostic biomarker of OC. Regarding the utility of serum exosomal miR-34a as a diagnostic biomarker, we are currently examining whether the expression of serum exosomal miR-34a differs between healthy women and OC patients. In addition, regarding the application of other serum miRNAs as biomarkers, since microarray profiling followed by qRT-PCR validation is currently acknowledged as the standard method for evaluating miRNA [12], we are conducting further comprehensive serum miRNA array analyses using serum exosomes.

A strength of this study is that it shows that miR-34a has potential as a useful biomarker not only for diagnosis but also prognosis. In addition, we demonstrate that the potential of miR-34a may be independent from the existing biomarker CA125. However, several limitations in this study should be acknowledged. In the present study, the potential advantages of biomarkers were shown. However, due to the small sample size, we were unable to fully investigate other factors, as would be required to develop a useful biomarker. In particular, we failed to show why differences among histological subtypes of OC occur. Furthermore, particularly in cases in elderly women, the management of OC should be personalized because of their differences from younger patients [43, 44]. Further research in a larger cohort is required to pursue the potential of serum miR-34a as an OC biomarker.

## Conclusions

Our results suggest that serum exosomal miR-34a could serve as potential biomarker of OC, and may be independent from CA125.

## Data Availability

The data used or analyzed are all contained within this published article.
